# Type 2 diabetes is independently associated with all-cause mortality secondary to ventricular tachyarrhythmias

**DOI:** 10.1186/s12933-018-0768-y

**Published:** 2018-09-10

**Authors:** Kathrin Weidner, Michael Behnes, Tobias Schupp, Jonas Rusnak, Linda Reiser, Armin Bollow, Gabriel Taton, Thomas Reichelt, Dominik Ellguth, Niko Engelke, Jorge Hoppner, Ibrahim El-Battrawy, Kambis Mashayekhi, Christel Weiß, Martin Borggrefe, Ibrahim Akin

**Affiliations:** 1 First Department of Medicine, University Medical Centre Mannheim (UMM), Faculty of Medicine Mannheim, University of Heidelberg, Theodor-Kutzer-Ufer 1-3, 68167 Mannheim, Germany; 2 European Center for AngioScience (ECAS) and DZHK (German Center for Cardiovascular Research) Partner Site Heidelberg/Mannheim, Mannheim, Germany; 3 Department of Diagnostic and Interventional Radiology, University Heidelberg, Heidelberg, Germany; 4 Department of Cardiology and Angiology II, University Heart Center Freiburg Bad Krozingen, Bad Krozingen, Germany; 5 Institute of Biomathematics and Medical Statistics, University Medical Center Mannheim (UMM), Faculty of Medicine Mannheim, Heidelberg University, Mannheim, Germany

**Keywords:** Ventricular tachyarrhythmias, Diabetes, Mortality, Death, Prognosis, Sudden cardiac death

## Abstract

**Objectives:**

The study sought to assess the prognostic impact of type 2 diabetes in patients presenting with ventricular tachyarrhythmias on admission.

**Background:**

Data regarding the prognostic outcome of diabetics presenting with ventricular tachyarrhythmias is limited.

**Methods:**

A large retrospective registry was used including all consecutive patients presenting with ventricular tachycardia (VT) and fibrillation (VF) on admission from 2002 to 2016. Patients with type 2 diabetes (diabetics) were compared to non-diabetics applying multivariable Cox regression models and propensity-score matching for evaluation of the primary prognostic endpoint of long-term all-cause mortality at 2 years. Secondary prognostic endpoints were cardiac death at 24 h, in-hospital death at index, all-cause mortality at 30 days, all-cause mortality in patients surviving index hospitalization at 2 years (i.e. “after discharge”) and rehospitalization due to recurrent ventricular tachyarrhythmias at 2 years.

**Results:**

In 2411 unmatched high-risk patients with ventricular tachyarrhythmias, diabetes was present in 25% compared to non-diabetics (75%). Rates of VT (57% vs. 56%) and VF (43% vs. 44%) were comparable in both groups. Multivariable Cox regression models revealed diabetics associated with the primary endpoint of long-term all-cause mortality at 2 years (HR = 1.513; p = 0.001), which was still proven after propensity score matching (46% vs. 33%, log rank p = 0.001; HR = 1.525; p = 0.001). The rates of secondary endpoints were higher for in-hospital death at index, all-cause mortality at 30 days, as well as after discharge, but not for cardiac death at 24 h or rehospitalization due to recurrent ventricular tachyarrhythmias.

**Conclusion:**

Presence of type 2 diabetes is independently associated with an increase of all-cause mortality in patients presenting with ventricular tachyarrhythmias on admission.

## Introduction

According to estimates by the international diabetes federation (IDF) 642 million people worldwide will suffer from diabetes mellitus type 2 in 2040. Therefore, diabetes represents a major burden to healthcare systems across the world [1, 2]. One of the most common causes of death in diabetics is sudden cardiac death (SCD) [3]. SCD accounts for 15–20% of all deaths in the Western world [4]. Furthermore, patients suffering from ventricular tachyarrhythmias and SCD are associated with poor outcome [5]. The identification of potential risk factors associated with increasing overall and cardiovascular mortality or SCD is of great medical interest.

The presence of type 2 diabetes constitutes a well-established cardiovascular risk factor. 70% of all hospitalizations in diabetics are due to vascular diseases and demand multidisciplinary therapies [6]. Type 2 diabetes affects all types of vessels regardless of vessel-size [7]. It induces microangiopathies such as diabetic retinopathy, nephropathy and neuropathy and macro-angiopathies including peripheral and coronary artery disease (CAD) [6]. In addition, prediabetes and type 2 diabetes are independently associated with the development of sub-clinical myocardial injury [8]. In turn, CAD and acute myocardial infarction (AMI) are the most common causes for the development of ventricular tachyarrhythmias and SCD [9, 10]. Over the last decades, type 2 diabetes has been evaluated as an independent risk factor for ventricular tachyarrhythmias and SCD [5, 11, 12]. However, it is still unclear whether type 2 diabetes may influence long-term prognosis of patients presenting with life-threatening ventricular tachyarrhythmias on admission.

Therefore, this study evaluates the secondary prognostic impact of type 2 diabetes in patients presenting with ventricular tachyarrhythmias on admission.

## Methods

### Study patients, design and data collection

The present study included retrospectively all patients presenting with ventricular tachyarrhythmias from 2002 until 2016 at one institution. All relevant clinical data related to the index event was documented using patients’ files, daily records, documentation from diagnostic examinations and laboratory values, electrocardiograms (ECG), device recordings, and all further information derived from the electronic hospital information system.

Ventricular tachyarrhythmias comprised ventricular tachycardia (VT) and fibrillation (VF), as defined by current international guidelines [5]. Sustained VT was defined by VT with a duration of more than 30 s or additional hemodynamic collapse within 30 s. Non-sustained VT are defined by less than 30 s. VT comprised wide QRS complexes (≥ 120 ms) at a rate greater than 100 beats/min [5]. Ventricular tachyarrhythmias were documented by 12-lead ECG, ECG tele-monitoring and implantable cardioverter defibrillators (ICD). In case of unstable course or during cardiopulmonary resuscitation (CPR) documentation was performed by external defibrillator monitoring. Documented VF was treated by external defibrillation and in case of prolonged instability with additional intravenous anti-arrhythmic drugs during CPR [5].

Further documented data contained baseline characteristics, prior medical history, prior medical treatment, length of index stay, detailed findings of laboratory values at baseline, data derived from all non-invasive or invasive cardiac diagnostics and device therapies. These included coronary angiography, electrophysiological examination, prior or newly implanted ICDs, pacemakers or cardiac contractility modulators (CCM), which were already implanted at index or at follow-up. Imaging modalities comprised echocardiography or cardiac magnetic resonance imaging (cMRI). The overall presence of an activated ICD summarizes the total sum of all patients with either a prior implanted ICD before admission, those undergoing new ICD implantation at index stay, as well as those with ICD implantation at the complete follow-up period after index hospitalization, referring to sole ICD, subcutaneous-ICD (s-ICD) and cardiac resynchronization therapy with defibrillator function (CRT-D). Pharmacological treatment was documented according to the discharge medication of patients surviving index hospitalization. Rates of overall ICDs and of pharmacological therapies are referred to the number of surviving patients being discharged from index hospitalization.

Documentation period lasted from index event until 2016. Documentation of all medical data was performed by independent cardiologists at the patients´ individual period of hospitalization blinded to final data analyses.

The present study is derived from an analysis of the “Registry of Malignant Arrhythmias and Sudden Cardiac Death-Influence of Diagnostics and Interventions (RACE-IT)” and represents a single-center registry including retrospectively consecutive patients presenting with ventricular tachyarrhythmias and SCD being acutely admitted to the University Medical Center Mannheim (UMM), Germany (clinicaltrials.gov identifier: NCT02982473) from 2002 until 2016. The registry was carried out according to the principles of the declaration of Helsinki and was approved by the medical ethics committee II of the Faculty of Medicine Mannheim, University of Heidelberg, Germany.

The medical center covers a general emergency department (ED) for emergency admission of traumatic, surgical, neurological and cardiovascular conditions. Interdisciplinary consultation is an inbuilt feature of this 24/7 service, and is connected to a stroke unit, four intensive care units (ICU) with extracorporeal life support and a chest pain unit (CPU) to alleviate rapid triage of patients. The cardiologic department itself includes 24 h catheterization and electrophysiologic laboratories, a hybrid operating room and telemetry units.

### Definition of study groups, inclusion and exclusion criteria

For the present analysis risk stratification was performed according to the presence of type 2 diabetes (diabetics) compared to non-diabetics following the guidelines of the international diabetes federation [13]. Diabetes mellitus was defined as HbA1c ≥ 6.5% (48 mmol/mol) or fasting plasma glucose level ≥ 7.0 mmol/L (≥126 mg/dl) or 2-h post-load plasma glucose level ≥ 11.1 mmol/L (≥200 mg/dL) [13, 14]. Patients with previously diagnosed and currently treated type 2 diabetes were included.

Overall exclusion criteria comprised patients with type 1 diabetes, patients with SCD without documentation of index ventricular tachyarrhythmias (VT or VF), and patients without complete follow-up data regarding mortality. Each patient was counted only once for inclusion when presenting with the first episode of ventricular tachyarrhythmias.

### Study endpoints

The primary prognostic endpoint was all-cause mortality at long-term follow-up. Secondary prognostic endpoints were early cardiac death at 24 h, in-hospital death at index, all-cause mortality at 30 days, all-cause mortality in patients surviving index hospitalization at 2 years (i.e. “after discharge”) and re-hospitalization due to recurrent ventricular tachyarrhythmias at 2 years. Early cardiac death was defined as occurring < 24 h after onset of ventricular tachyarrhythmias or an assumed unstable cardiac condition on index admission [5].

Overall follow-up lasted until 2016. All-cause mortality was documented using our electronic hospital information system and by directly contacting state resident registration offices (“bureau of mortality statistics”) across Germany. Identification of patients was verified by place of name, surname, day of birth and registered living address. Lost to follow-up rate was 1.7% (n = 48) regarding survival until the end of the follow-up period.

### Statistical methods

Quantitative data are presented as mean ± standard error of mean (SEM), median and interquartile range (IQR), and ranges depending on the distribution of the data and were compared using the Student’s *t* test for normally distributed data or the Mann–Whitney *U* test for nonparametric data. Deviations from a Gaussian distribution were tested by the Kolmogorov–Smirnov test. Spearman’s rank correlation for nonparametric data was used to test univariate correlations. Qualitative data are presented as absolute and relative frequencies and compared using the Chi^2^ test or the Fisher’s exact test, as appropriate.

Firstly, overall data of consecutive patients on admission are given for the entire unmatched cohort in order to present the real-life character of health-care supply at our institution in between 2002 and 2016. Here, multivariable Cox regression models were applied for the evaluation of the primary prognostic endpoint within the total study cohort for diabetics compared to non-diabetics. Then uni- and multivariable Cox regression models were applied for the primary prognostic endpoint for diabetes in the sub-groups of males, females, age above or below 70 years, VT, VF, chronic kidney disease, acute myocardial infarction (AMI), (non-)ST segment elevation myocardial infarction (NSTEMI and STEMI), CAD, non-CAD, left ventricular ejection fraction (LVEF) above or below 55%, overall ICD, primary and secondary preventive ICD, non-ICD patients. Multivariable Cox regression models were adjusted for the following covariables: age, gender, chronic kidney disease, CAD, CPR, AMI, LVEF < 55%, index ventricular tachyarrhythmia (i.e., VT/VF) and overall ICD.

Secondly, propensity score matching was applied. There is a relevant and increasing demand from patients, clinicians and within the health care system in general for growing evidence from non-randomized studies. There are simply too many medically relevant questions and hypotheses, which will never be investigated within randomized controlled trials due to several reasons (i.e. funding, recruitment, difficult study settings, high-risk patients, etc.). Therefore, we felt that the method of propensity score matching would be a reasonable additional statistical method beside multivariable Cox regression models for the purpose of the present study evaluating the prognostic impact of diabetes in high-risk patients presenting with ventricular tachyarrhythmias on admission. These high-risk patients are usually excluded from randomized controlled trials (RCT). In a RCT patients with or without a specific treatment would have a 50% chance to be treated and balanced measured and unmeasured baseline characteristics would be expected. However, patients with different disease entities may not be randomized in real-life (such as diabetics vs. non-diabetics) due to different pathophysiologies and treatment recommendations. An observational study usually recruits consecutive real-life patients without randomization resulting in varying chances between 0% and 100% to receive imbalances in baseline characteristics and treatments. Therefore, differences of outcomes in specific disease groups might be explained by heterogeneous distribution of baseline characteristics and applied therapies. To further reduce this selection bias, we used 1:1 propensity-scores for diabetics vs. non-diabetics to assemble matched cohorts, in which patients would be well-balanced regarding all measured baseline characteristics. 1:1 propensity score matching was performed including the entire study cohort, applying a non-parsimonious multivariable logistic regression model using diabetics as the dependent variables [15, 16].

Propensity scores were created according to the presence of the following independent variables: age, gender, chronic kidney disease, CAD, acute myocardial infarction, LVEF, CPR, index ventricular tachyarrhythmia (i.e., VT/VF) and overall ICD. Based on the propensity score values counted by logistic regression, for each diabetic one non-diabetic in the control group with a similar propensity score value was found (accepted difference of propensity score values < 5%). Uni-variable stratification was performed using the Kaplan–Meier method with comparisons between groups using uni-variable hazard ratios (HR) given together with 95% confidence intervals, according to the presence of diabetics and non-diabetics within the propensity-matched cohorts.

Follow-up periods for evaluation of long-term all-cause mortality were set at 2 years according to the median survival time of diabetic patients to guarantee complete follow-up of at least 50% of patients. Patients not meeting long-term follow-up were censored.

The result of a statistical test was considered significant for p < 0.05, p values ≤ 0.1 were defined as a statistical trend. SAS, release 9.4 (SAS Institute Inc., Cary, NC, USA) and SPSS (Version 25, IBM Armonk, New York, USA) were used for statistics.

## Results

### Entire, unmatched real-life cohort

In the entire unmatched real-life cohort of 2411 consecutive patients presenting with ventricular tachyarrhythmias on admission the prevalence of type 2 diabetes was 25%. As shown in Table 1 (left columns) rates of VT and VF were comparable between diabetics and non-diabetics (57% vs. 43%) and most patients were males (72%). Diabetics were older (median 71 vs. 67 years) and had higher rates of arterial hypertension, hyperlipidaemia, prior heart failure, prior AMI, prior CAD, atrial fibrillation, chronic kidney disease, stroke and CPR, whereas rates of AMI at index were similar. Furthermore, diabetics had higher rates of coronary 3 vessel disease with chronic total occlusions and higher rates of LVEF < 35%.

**Table 1:** Study population

Characteristic	Before matching (n = 2411)			After matching (n = 894)		
	Non-diabetics (n = 1798; 75%)	Diabetics (n = 613; 25%)	p value	Non-diabetics (n = 447; 50%)	Diabetics (n = 447; 50%)	p value
Ventricular tachyarrhythmias, n (%)						
Ventricular tachycardia	1006 (56)	350 (57)	0.622	275 (62)	272 (61)	0.837
Monomorphic	973 (97)	339 (97)	0.901	267 (97)	262 (96)	0.615
Polymorphic	33 (3)	11 (3)		8 (3)	10 (4)	
Ventricular fibrillation	792 (44)	263 (43)	0.622	172 (39)	175 (39)	0.837
Age, median (range)	67 (14–97)	71 (33–95)	*0.001*	70 (21–94)	71 (33–91)	*0.040*
Male gender, n (%)	1288 (72)	440 (72)	0.946	348 (78)	331 (74)	0.183
Cardiovascular risk factors, n (%)						
Arterial hypertension	891 (50)	464 (76)	*0.001*	287 (64)	351 (79)	*0.001*
Hyperlipidemia	448 (25)	210 (34)	*0.001*	152 (34)	164 (37)	0.401
Smoking	478 (27)	161 (26)	0.876	133 (30)	128 (29)	0.713
Cardiac family history	188 (11)	34 (6)	*0.001*	53 (12)	26 (6)	*0.001*
Comorbidities, n (%)						
Prior heart failure	355 (20)	196 (32)	*0.001*	143 (32)	172 (39)	*0.042*
Prior coronary artery disease	608 (34)	336 (55)	*0.001*	248 (56)	270 (60)	0.136
Prior myocardial infarction	363 (20)	180 (29)	*0.001*	147 (33)	150 (34)	0.831
Valvular heart disease	138 (8)	74 (12)	*0.001*	46 (10)	61 (14)	0.122
Acute myocardial infarction	507 (28)	180 (29)	0.581	123 (28)	128 (29)	0.710
STEMI	184 (10)	52 (9)	0.208	29 (7)	38 (9)	0.253
NSTEMI	323 (18)	128 (21)	0.110	94 (21)	90 (20)	0.741
Non-ischemic cardiomyopathy	96 (5)	27 (4)	0.364	32 (7)	27 (6)	0.501
Atrial fibrillation	492 (27)	223 (36)	*0.001*	155 (35)	170 (38)	0.297
Stroke (ischemic or hemorrhage)	42 (2)	30 (5)	*0.001*	12 (3)	20 (5)	0.150
Chronic kidney disease	801 (46)	387 (64)	*0.001*	25 (56)	27 (62)	*0.048*
Cardiopulmonary resuscitation	831 (52)	425 (57)	*0.001*	175 (39)	198 (44)	0.119
In hospital	310 (17)	169 (28)	*0.001*	77 (17)	109 (24)	*0.008*
Out of hospital	521 (29)	139 (23)	*0.001*	98 (22)	89 (20)	0.459
Coronary angiography, overall, n (%)	1055 (59)	362 (59)	0.870	291 (65)	295 (66)	0.778
Coronary artery disease	742 (41)	305 (50)	*0.001*	233 (52)	249 (56)	0.155
None	313 (30)	57 (16)	*0.001*	58 (20)	46 (16)	0.169
1-vessel	244 (23)	82 (23)	0.853	74 (25)	66 (22)	0.386
2-vessel	249 (24)	86 (24)	0.952	73 (25)	73 (25)	0.924
3-vessel	249 (24)	137 (38)	*0.001*	86 (30)	110 (37)	*0.047*
Chronic total occlusion	190 (18)	100 (28)	*0.001*	70 (24)	82 (28)	0.301
Presence of CABG	110 (10)	73 (20)	*0.001*	49 (17)	66 (22)	0.092
Percutaneous coronary intervention	479 (45)	163 (45)	0.902	113 (39)	123 (42)	0.480
Left ventricular ejection function, n (%)						
LVEF ≥ 55%	431 (24)	101 (16)	*0.001*	102 (23)	100 (22)	0.873
LVEF 54–35%	449 (25)	160 (26)	0.579	156 (35)	139 (31)	0.227
LVEF < 35%	427 (24)	211 (34)	*0.001*	189 (42)	208 (47)	0.201
Not documented	491 (27)	141 (23)	–	–	–	–
Electrophysiological examination, n (%)	481 (27)	111 (18)	*0.001*	128 (30)	88 (20)	*0.001*
Induced ventricular tachycardia						
Inferior	11 (2)	2 (2)	0.753	3 (2)	2 (2)	0.973
Apical	152 (32)	40 (36)	0.368	42 (33)	35 (40)	0.294
Septal	18 (4)	1 (0.9)	0.126	6 (5)	0 (0)	0.083
Lateral	5 (1)	4 (4)	0.069	0 (0)	3 (3)	0.066
Left ventricular	23 (5)	4 (4)	0.592	6 (5)	3 (3)	0.741
Right ventricular	162 (34)	43 (39)	0.313	53 (41)	38 (41)	0.795
LVOT	7 (2)	3 (3)	0.358	3 (2)	2 (2)	1.000
RVOT	84 (18)	15 (14)	0.315	21 (16)	14 (16)	1.000
Ablation of ventricular tachycardia, n (%)	117 (7)	13 (2)	*0.001*	31 (7)	9 (2)	*0.001*
Patients at discharge, n (%)	1296 (72)	402 (66)	*0.002*	357 (80)	322 (72)	*0.001*
Presence of ICD overall, n (%)	643 (50)	217 (54)	0.126	214 (60)	190 (59)	0.816
ICD	572 (89)	189 (87)	0.458	192 (91)	165 (87)	0.237
s-ICD	24 (4)	7 (3)	0.729	0 (0)	7 (4)	1.000
CRT-D	47 (7)	21 (10)	0.264	20 (9)	18 (10)	0.989
Bipolar electrodes	17 (36)	6 (29)	0.541	8 (40)	5 (28)	0.428
Multipolar electrodes	30 (64)	15 (71)		12 (60)	13 (72)	
Primary prevention	266 (41)	109 (50)	*0.023*	97 (45)	98 (52)	0.209
Secondary prevention	377 (59)	108 (50)		117 (55)	92 (48)	
Medication at discharge, n (%)						
Beta-blocker	1010 (78)	336 (84)	*0.014*	311 (87)	279 (87)	0.857
ACEi/ARB	904 (70)	323 (80)	*0.001*	249 (82)	266 (83)	0.930
Statin	734 (57)	283 (71)	*0.001*	256 (72)	234 (73)	0.780
Digitalis	133 (10)	69 (17)	*0.001*	50 (14)	59 (18)	0.126
Amiodarone	169 (13)	88 (22)	*0.001*	66 (19)	69 (21)	0.338
Sotalol	10 (0.8)	5 (1)	0.376	2 (0.6)	4 (1)	0.430
Vitamin K antagonist	225 (17)	88 (22)	*0.038*	88 (25)	74 (23)	0.611
NOAC	29 (2)	9 (2)	0.992	7 (2)	6 (2)	0.926
Low molecular heparin	95 (7)	40 (10)	0.089	32 (9)	31 (10)	0.766

*ACE* angiotensin converting enzyme, *ARB* angiotensin II receptor blocker, *CABG* coronary artery bypass grafting, *CRT-D* cardiac resynchronization therapy with defibrillator, *DM* type 2 diabetes mellitus, *ICD* implantable cardioverter defibrillator, *LVEF* left ventricular ejection fraction, *LVOT/RVOT* left/right ventricular outflow tract, *s-ICD* subcutaneous ICD, *NOAC* novel oral anticoagulant, *(N)STEMI* (non) ST segment elevation myocardial infarction

Rates of electrophysiological examination were higher in non-diabetics compared to diabetics (27% vs. 18%). Morphologies of induced VT were similar in both groups. Non-diabetics underwent ablation therapy for VT more often (7% vs. 2%) with a median LVEF 42% (IQR 22–57%) in these patients (Table 1, left columns). Overall rates of ICDs were similar in diabetics and non-diabetics (50% vs. 54%), respectively for CRT-D recipients without further differences in bipolar and multipolar electrodes. ICD implantation was indicated more often for secondary prevention in non-diabetics and for primary prevention in diabetics (Table 1, left columns).

Diabetics were more often treated with beta blockers, angiotensin converting enzyme inhibitors (ACEi), angiotensin receptor blockers (ARB), statins, digitalis and amiodarone and vitamin K antagonists. In contrast, rates of sotalol, novel oral anticoagulants (NOAC) and low-molecular heparin were similar (Table 1, left columns). Regarding antidiabetic medication (Table 2), most diabetics were treated with insulins, followed by metformin and sulfonylurea. Glitazone, dipeptidyl peptidase-4 (DPP-4) inhibitors and sodium/glucose cotransporter (2) (SGLT(2)) -inhibitors were administered rarely (less than 4%).

**Table 2:** Antidiabetic medication in diabetics with ventricular tachyarrythmias

	Before matching (n = 613; 25%)	After matching (n = 447; 50%)
Insulin, long-acting	134 (33)	109 (34)
Insulin, short-acting	61 (15)	50 (16)
Sulfonylurea	68 (17)	59 (18)
Metformin	61 (15)	50 (16)
Glitazone	2 (0.5)	2 (0.6)
DPP4 inhibitor	14 (4)	12 (4)
SGLT(2)-inhibitor	1 (0.2)	1 (0.3)

*DPP4* dipeptidyl peptidase-4, *SGLT(2)* sodium/glucose cotransporter 2

**Fig. 1:**
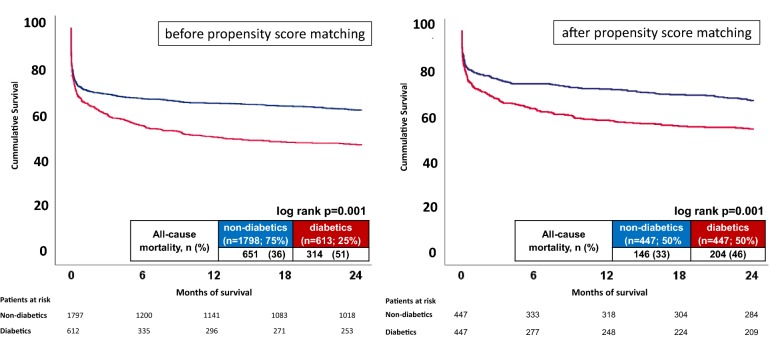
Kaplan Meier survival curves demonstrating higher long-term all-cause mortality before (left panel) and after propensity score matching (right panel) in diabetics presenting with ventricular tachyarrhythmias

Figure 1 (left panel) illustrates significantly higher rates of the primary endpoint of all-cause mortality at 2 years after presenting with ventricular tachyarrhythmias on hospital admission (51% vs. 36%, log rank p = 0.001; HR = 1.513, 95% CI 1.322–1.731; p = 0.001). Furthermore, diabetics were associated with higher rates of secondary endpoints, including in-hospital death, all-cause mortality at 30 days and after discharge, whereas cardiac death at 24 hours and rehospitalization due to recurrent ventricular tachyarrhythmias were similar in both groups (Table 6, right columns).

Multivariable Cox regression analyses within the entire unmatched “real-life” cohort revealed diabetics being significantly associated with the primary prognostic endpoint of long-term all-cause mortality at 2 years (HR = 1.209; 95% CI 1.010–1.447; p = 0.039) (Table 3). Diabetics sustained significant impact on long-term all-cause mortality in the sub-groups of males, age > 70 years, VT, AMI, NSTEMI, overall CAD, multi-vessel CAD and ICD-recipients, respectively with indication for primary prevention (Table 4). Notably, insulin dependent diabetics were associated with increased long-term all-cause mortality, whereas treatment with metformin and other oral antidiabetic drugs in diabetics revealed prognostic benefit **(**unadjusted hazard ratios, Table 5). In CRT-D patients no differences were seen in between patients with multipolar compared to bipolar ICD electrodes (non-diabetics: 18% vs. 14% p = 0.836; diabetics: 26% vs. 17% p = 0.417; data not shown).

**Table 3:** Unmatched uni- and multivariable hazard ratios to predict the primary prognostic endpoint of long-term all-cause mortality at 2 years (n = 2422)

	n (%)	Univariable			Multivariable		
		HR	95% CI	p value	HR	95% CI	p value
Age > 70 years	1057 (44)	1.924	1.714–2.210	*0.001*	1.560	1.305–1.866	*0.001*
Male gender	1728 (72)	0.945	0.822–1.086	0.428	1.299	1.069–1.579	*0.008*
Chronic kidney disease	1188 (49)	3.637	3.136–4.218	*0.001*	2.792	2.293–3.400	*0.001*
Acute myocardial infarction	687 (28)	1.422	1.245–1.624	*0.001*	0.719	0.587–0.881	*0.001*
LVEF < 55%	1247 (52)	1.622	1.332–1.976	*0.001*	1.861	1.509–2.294	*0.001*
Coronary artery disease	1490 (62)	0.959	0.842–1.091	0.521	0.938	0.766–1.147	0.531
Cardiopulmonary resuscitation	1256 (52)	2.312	2.146–2.491	*0.001*	1.841	1.657–2.045	*0.001*
Presence of an ICD, overall	860 (36)	0.207	0.172–0.248	*0.001*	0.245	0.196–0.306	*0.001*
Diabetes	613 (25)	1.513	1.322–1.731	*0.001*	1.209	1.010–1.447	*0.039*

*CI* confidence interval, *CKD* chronic kidney disease, *HR* hazard ratio, *ICD* implantable cardioverter defibrillator, *LVEF* left ventricular ejection fraction

Level of significance p < 0.05; statistical trend p < 0.1

**Table 4:** Unmachted univariable and multivariable hazard ratios for the association of diabetics with the primary prognostic endpoint of long-term all-cause mortality at 2 years in pre-specified sub-groups

	n (%)	Univariable			Multivariable^a^		
		HR	95% CI	p value	HR	95% CI	p value
Total cohort	2422 (100)	1.513	1.322–1.731	*0.001*	1.209	1.010–1.447	*0.039*
Females	684 (28)	1.472	1.144–1.895	*0.003*	0.881	0.606–1.280	0.506
Males	1738 (72)	1.528	1.303–1.792	*0.001*	*1.328*	*1.080–1.632*	*0.007*
Age < 70 years	1365 (56)	1.564	1.258–1.945	*0.001*	1.189	0.950–1.488	0.131
Age > 70 years	1057 (44)	1.245	1.047–1.481	*0.013*	*1.369*	*1.019–1.840*	*0.037*
Ventricular tachycardia	1364 (56)	2.030	1.658–2.486	*0.001*	*1.351*	*1.041–1.754*	*0.024*
Ventricular fibrillation	1058 (44)	1.222	1.018–1.466	*0.032*	1.039	0.803–1.344	0.771
Chronic kidney disease	1194 (49)	1.267	1.084–1.481	*0.003*	1.206	0.984–1.478	0.071
No chronic kidney disease	1228 (51)	1.564	1.170–2.089	*0.002*	1.206	0.819–1.776	0.342
Acute myocardial infarction	691 (29)	1.291	1.020–1.634	*0.033*	*1.569*	*1.144–2.150*	*0.005*
ST segment elevation myocardial infarction	237 (10)	1.155	0.723–1.846	0.546	1.441	0.711–2.922	0.311
Non segment elevation myocardial infarction	454 (19)	1.309	0.995–1.721	0.054	*1.692*	*1.180–2.425*	*0.004*
Coronary artery disease, overall	1490 (62)	2.113	1.687–2.646	*0.001*	*1.327*	*1.078–1.634*	*0.008*
Coronary multivessel disease	721 (30)	1.221	0.953–1.563	0.114	*1.516*	*1.110–2.070*	*0.009*
No coronary artery disease	932 (39)	1.306	1.103–1.547	*0.002*	0.943	0.653–1.362	0.753
LVEF ≥ 55%	532 (22)	2.494	1.725–3.606	*0.001*	1.248	0.835–1.866	0.280
LVEF < 55%	1247 (51)	1.600	1.320–1.941	*0.001*	1.192	0.975–1.457	0.086
Implantable cardioverter defibrillator	860 (36)	2.046	1.447–2.893	*0.001*	*1.859*	*1.272–2.715*	*0.001*
Primary prevention	397 (16)	2.736	1.688–4.436	*0.001*	*1.902*	*1.116–3.242*	*0.018*
Secondary prevention	463 (20)	1.577	0.957–2.600	0.074	1.651	0.940–2.900	0.081
No implantable cardioverter defibrillator	1562 (64)	1.466	1.266–1.698	*0.001*	1.086	0.885–1.332	0.430

*CI* confidence interval, *HR* hazard ratio, *LVEF* left ventricular ejection faction

Level of significance p < 0.05; statistical trend p < 0.1

^a^Multivariable models were adjusted for age, gender, chronic kidney disease, ventricular tachyarrhythmias, LV dysfunction, CPR, AMI and presence of an activated ICD (overall)

**Table 5:** Unmatched univariable hazard ratios for the association of anti-diabetic therapies with the primary prognostic endpoint of all-cause mortality at 2 years

	n (%)	Univariable		
		HR	95% CI	p value
Insulin dependent	148 (37)	1.603	1.087–2.362	*0.017*
Metformin	61 (15)	0.256	0.104–0.628	*0.003*
Oral antidiabetic drugs	84 (21)	0.493	0.270–0.900	*0.021*

*CI* confidence interval, *HR* hazard ratio

Level of significance p < 0.05; statistical trend p < 0.1

### Propensity matched cohort

After applying propensity score matching for the comparison of diabetics vs. non-diabetics (447 matched pairs) comparable rates were achieved for ventricular tachyarrhythmias at index, gender, AMI, atrial fibrillation, CPR, overall CAD, LVEF, overall ICD and medication at discharge. There were only slight differences left for age (71 vs. 70 years), stroke (62% vs. 56%), 3-vessel CAD (37% vs. 30%) and chronic kidney disease (62% vs. 56%) between diabetics and non-diabetics (Table 1, right columns).

Figure 1 (right panel) illustrates significantly higher rates of the primary endpoint of all-cause mortality at 2 years even after propensity score matching in patients presenting with ventricular tachyarrhythmias on hospital admission (46% vs. 33%, log rank p = 0.001; HR = 1.525, 95% CI 1.234–1.885; p = 0.001). Furthermore, diabetics were associated with higher rates of secondary endpoints, including in-hospital death, all-cause mortality at 30 days and after discharge, whereas cardiac death at 24 hours and rehospitalization due to recurrent ventricular tachyarrhythmias were similar in both groups, even after propensity score matching (Table 6, right columns).

**Table 6:** Primary and secondary endpoints

	Before matching (n = 2411)			After matching (n = 894)		
	Non-diabetics (n = 1798; 75%)	Diabetics (n = 613; 25%)	p value	Non-diabetics (n = 447; 50%)	Diabetics (n = 447; 50%)	p value
Primary endpoint, n (%)						
All cause-mortality, at 2 years	651 (36)	314 (51)	*0.001*	146 (33)	204 (46)	*0.001*
Secondary endpoints, n (%)						
Cardiac death, at 24 h	308 (17)	119 (19)	0.201	47 (11)	63 (14)	0.103
All cause-mortality, at 30 days	488 (27)	202 (33)	*0.006*	87 (19)	120 (27)	*0.009*
In-hospital death, at index	502 (28)	211 (34)	*0.002*	91 (20)	126 (28)	*0.006*
All-cause mortality, after discharge	149 (8)	103 (17)	*0.001*	55 (12)	78 (17)	*0.050*
Rehospitalization for ventricular tachyarrhythmias	120 (9)	35 (9)	0.737	32 (9)	25 (8)	0.574
Follow up times						
Hospitalization total; days [median (IQR)]	10 (4–18)	13 (6–23)	*0.008*	12 (7–23)	14 (7–24)	0.277
ICU time; days [median (IQR)]	2 (0–7)	4 (1–9)	*0.001*	3 (0–8)	4 (1–9)	*0.005*
Follow-up; days [mean; median (range)]	1488; 1087; (0–5106)	1005; 299 (0–5106)	*0.001*	1650; 1434 (0–5004)	1158; 541 (0–5106)	*0.008*

*ICU* invasive care unit, *IQR* interquartile range

## Discussion

The present study evaluates the prognostic impact of type 2 diabetes in consecutive high-risk patients presenting with ventricular tachyarrhythmias on admission.

This real-world data suggests that high-risk patients presenting with ventricular tachyarrhythmias on admission reveal higher long-term all-cause mortality in the presence of diabetes. Respectively, increasing rates of secondary endpoints, including in-hospital death at index, all-cause mortality at 30 days and long-term mortality in patients surviving index hospitalization were observed in diabetics compared to non-diabetics. Prognostic differences of long-term all-cause mortality for diabetics were verified in several subgroups even after multivariable adjustment including males, age > 70 years, VT, AMI, NSTEMI, overall CAD, multi-vessel CAD and ICD-recipients, respectively with indication for primary prevention. This study identifies the presence of type 2 diabetes as a robust predictor of all-cause mortality in patients presenting with ventricular tachyarrhythmias straight from the admission scenario, whereas early cardiac death at 24 h and rehospitalization due to recurrent ventricular tachyarrhythmias were not affected by the presence of type 2 diabetes.

The presence of diabetes is an established cardiovascular risk factor, which is associated with increasing rates of myocardial infarction, stroke and both all-cause and cardiovascular mortality in the general population [17, 18]. Diabetics without prior myocardial infarction reveal a comparable risk as non-diabetics with prior myocardial infarction regarding future occurrence of myocardial infarction and present with higher rates of cardiac rehospitalisation [17, 19]. The increased risk of mortality related to diabetes has been demonstrated in patients suffering from CAD, especially multi-vessel-CAD, where coronary artery bypass grafting (CABG) was proven as the best type of coronary revascularization in terms of mortality reduction compared to PCI [20–23]. The vascular system of diabetics is affected by oxidative stress, endothelial dysfunction, atherogenesis and vascular remodeling alleviating coronary atherosclerosis, but also diabetic neuropathy [24, 25]. Diabetic neuropathy promotes silent myocardial ischemia, which in turn may mimic typical angina and further signs of myocardial infarction [26]. Changes in lifestyle and effective antidiabetic medical therapy may significantly reduce levels of Hba1c below the recommended treatment target of 7% in diabetics, which may at the same time attenuate CAD development and improve prognosis [18, 27, 28]. In clear contrast, the prognostic impact of diabetes in CAD patients presenting with ventricular tachyarrhythmias has rarely been investigated. In this respect, the present study delivers novel evidence, demonstrating the adverse prognostic impact of diabetes on long-term all-cause mortality at 2 years even in the subgroups of AMI, NSTEMI, overall CAD and multi-vessel CAD.

Diabetic cardiomyopathy is another consecutive comorbidity, which is defined as concomitant heart failure in the absence of arterial hypertension and CAD [29]. The metabolic milieu in diabetics is characterized by hyperglycemia, increased fatty acids, triacylglycerols, inflammatory cytokines and hyperinsulinemia. These alterations sustain structural changes of the myocardium due to loss of cardiolipins with consecutive intra-myocardial lipid-accumulation [30, 31]. Modern concepts of optimal therapy monitoring as well as of prediction of heart failure deterioration and rehospitalization, especially in diabetic ICD recipients comprise the introduction of tele-monitoring systems. Their prognostic benefits in terms of reduction of all-cause and cardiovascular mortality is debated [32]. The present study has not implied any kind of tele-monitoring, which may in future improve further the management of diabetic heart failure patients even in the presence of ventricular tachyarrhythmias.

The metabolic syndrome is associated with co-existing obesity, hyperlipidemia, arterial hypertension and (pre-) diabetes [33, 34]. Obese patients are associated with a two to three-fold higher risk for CAD than non-obese patients alongside an increased risk of mortality [35, 36]. Whether obese patients are also associated with an increased risk for ventricular tachyarrhythmias compared to non-obese patients is controversial [34, 37]. Both obese and non-obese patients with mild stages of systolic heart failure reveal a comparable risk of ventricular tachyarrhythmias and comparable benefit from CRT therapy [34]. The metabolic syndrome itself may impact cardiac electrophysiological alterations and response to CRT-D therapy [33], which may be reflected by alterations of thresholds of CRT-D leads parameters [33] leading to impaired response even at long-term follow-up [33, 38, 39]. Accordingly, electrocardiographic changes are found in diabetics potentially related to transmural dispersion of repolarization in terms of QRS and QT prolongation compared to non-diabetics [24]. Besides simple ECG recordings [40], novel biomarkers reflecting diabetes, heart failure and the metabolic syndrome, such as natriuretic peptides and neutrophil gelatinase-associated lipocalin (NGAL) [41–46] may reveal the potential to improve risk-stratification in terms of prediction of all-cause and cardiovascular mortality in high-risk patients with ventricular tachyarrhythmias in future [45, 46].

Age has been identified as a significant risk factor for cardiovascular-related morbidity and mortality [47]. In the present study diabetics > 70 years were associated with a 1.3-fold higher risk of death at 2 years, where concomitant heart failure is usually present. It may be speculated whether the prognostic benefit of an ICD therapy may become overt in the elderly diabetic, since they may die from increasing co-morbidities without effective utilization of their device [48, 49]. Whether this may concern diabetics with documented episodes of ventricular tachyarrhythmias awaits further research.

Novel oral antidiabetics were shown to decrease cardiovascular mortality [21, 22]. The biguanide metformin and the sodium/glucose cotransporter 2 (SGLT2) inhibitor empagliflozin were associated with a significant reduction of all-cause mortality and cardiovascular events in diabetics [36, 50]. The prognostic impact of incretin and its analogs on mortality or cardiovascular events is currently debated, since no reduction of major adverse cardiac events was demonstrated in patients with and without CAD by the dipeptidyl peptidase-4 (DPP-4) inhibitor sitagliptin [51], whereas observational studies demonstrated adverse prognosis in STEMI/NSTEMI patients without incretin therapy [21, 22]. Evaluation of pharmacological effects of novel antidiabetic drugs was beyond the scope of the present study, where only a minor number of patients were treated by these therapeutics. The univariable prognostic benefits in terms of mortality reduction seen for metformin and overall oral antidiabetics do not allow reliable conclusions for patients with ventricular tachyarrhythmias on admission.

In summary, this study demonstrates increasing all-cause mortality at 2-years in diabetics compared to non-diabetics presenting with ventricular tachyarrhythmias on admission. Respectively, increasing rates of secondary endpoints, including in-hospital death at index, all-cause mortality at 30 days and long-term mortality in patients surviving index hospitalization were seen in diabetics compared to non-diabetics. Therefore, the presence of diabetes represents a robust predictor of all-cause mortality in patients presenting with ventricular tachyarrhythmias, as proven also in several sub-groups. The present results add to the knowledge of previous diabetes studies highlighting the need for a better risk stratification of high risk diabetics presenting with ventricular tachyarrhythmias focussing on improvement of effective diagnostics and therapies.

### Study limitations

This observational and retrospective registry-based analysis reflects a realistic picture of consecutive health-care supply of high-risk patients presenting with ventricular tachyarrhythmias. Lost to follow-up rate regarding the evaluated endpoint of all-cause mortality was minimal. Although heterogeneity within the study population was controlled by a stepwise statistical approach including multivariable adjustment and propensity score, some minor differences were still seen e.g. for chronic kidney disease. This may reflect the presence of diabetic nephropathy in this cohort. Patients not surviving out of hospital cardiac arrest were not transferred to the heart centre and therefore were not included in this study. All clinical data was documented reliably by individual cardiologists, who were blinded to the final analysis. They documented their results during routine clinical care, which alleviates the use of an independent clinical event committee. The present study did not assess data on body mass index (BMI), digitalis, amiodarone, sotalol, continuous monitoring systems or novel cardiac biomarkers. Future randomized or even multicenter studies may reevaluate the results of the present study.

## Conclusions

This real-world data suggests that high-risk patients presenting with ventricular tachyarrhythmias on admission are associated with increasing all-cause mortality in the presence of diabetes.

## Abbreviations

AMI: acute myocardial infarction
CABG: coronary artery bypass grafting
CAD: coronary artery disease
COPD: chronic obstructive pulmonary disease
CPR: cardiopulmonary resuscitation
CTO: chronic total occlusion
DM: diabetes mellitus
ECG: electrocardiogram
ICD: implantable cardioverter defibrillator
LVEF: left ventricular ejection fraction
NSTEMI: non-ST segment elevation myocardial infarction
SCD: sudden cardiac death
STEMI: ST segment elevation myocardial infarction
VF: ventricular tachycardia
VT: ventricular tachycardia
